# Editorial: Targeting the Chemoattractant System in Inflammation

**DOI:** 10.3389/fphar.2021.744290

**Published:** 2021-08-16

**Authors:** Tadashi Hosoya, Dunai Cordelia, Benedict D. Michael, Chie Miyabe, Jun Nagai, Thomas T. Murooka, Yoshishige Miyabe

**Affiliations:** ^1^ Department of Rheumatology, Tokyo Medical and Dental University (TMDU), Liverpool, United Kingdom; ^2^ Clinical Infection Microbiology and Immunology, Institute of Infection Ecology and Veterinary Sciences, University of Liverpool, Liverpool, United Kingdom; ^3^ NIHR HPRU for Emerging and Zoonotic Infection, Liverpool, United Kingdom; ^4^ The Walton Centre NHS Foundation Trust, Liverpool, United Kingdom; ^5^ Division of Dermatology, Tokyo Women’s Medical University, Chiba, Japan; ^6^ Department of Medicine, Harvard Medical School, Boston, MA, United States; ^7^ Division of Allergy and Clinical Immunology, Brigham and Women’s Hospital, Boston, MA, United States; ^8^ Department of Immunology, University of Manitoba, Winnipeg, MB, Canada; ^9^ Department of Cell Biology, Nippon Medical School, Institute for Advanced Medical Sciences, Tokyo, Japan

**Keywords:** chemokine, complement, lipid mediator, immunology, inflammation

## Introduction

In autoimmune diseases and infectious diseases, the recruitment of leukocytes from blood vessels to the target tissue is the fundamental component of inflammation. Thus, the control of leukocytes’ entry into the tissue represents a major point to which new therapeutics could be developed to attenuate inflammatory diseases (Miyabe et al., 2019a). The process of leukocyte migration is regulated by the chemoattractant systems including chemokines, complement components and lipid mediators and their receptors (Miyabe et al., 2017a; Miyabe et al., 2019a). Our recent *in vivo* imaging studies have discovered a new function of complement component C5a and atypical complement receptor C5aR2 in immune complex-induced arthritis (Miyabe et al., 2017b; Miyabe et al., 2019b). Moreover, we have demonstrated that the chemokine is a key initiator of leukocytes recruitment into the coronary artery (Miyabe et al., 2019c) and brain during cerebral malaria and herpes encephalitis (Sorensen et al., 2018; Michael et al., 2020).

Numerous chemoattractant systems have been shown to play important roles in the recruitment of leukocytes into inflamed tissues in animal models of inflammatory diseases. In contrast, clinical trials of chemokine-targeting therapy in rheumatic diseases such as rheumatoid arthritis have generally ended with disappointing results (Miyabe et al., 2019a). This might be due to relevant chemoattractant receptors needing to be blocked at all times for therapeutic effect. In addition, there is functional overlap between many chemoattaractant systems involved in leukocytes trafficking, and inhibition of a single chemoattractant system might not be sufficient to completely suppress leukocytes recruitment (Miyabe et al., 2019a). More effective approaches might combine targeting multiple chemoattractants and/or their receptors, as indicated by our studies in mouse models (Angelini et al., 2018). To develop more effective therapies for targeting chemoattractants, we should accumulate knowledge about the pattern of chemoattractants and their receptors and clarify the function of chemoattractant systems in individual diseases.

This research topic includes various articles in the field: three original articles and one review article. Two articles aimed at novel drug development for targeting chemokine and inflammatory cytokines. One article revealed a mode of action in monosodium urate (MSU)- induced inflammation. The review summarized the contribution of the chemokine system in the three epidemic coronavirus infections, including Severe Acute Respiratory Syndrome coronavirus-2(SARS-CoV2), SARS-CoV1, and Middle East Respiratory Syndrome-coronavirus (MERS).

## Identifying Novel Therapeutic Agents Targeting Chemokine or Inflammatory Cytokines

Hosoya et al. identified a novel therapeutic candidate based on the cell-based high throughput screening (HTS). CXCL8 secreted from THP-1 cells, a human monocyte cell line, was selected as a readout of HTS. To enrich the candidates, the authors chose compounds with NF-κB inhibition similar to glucocorticoids (GCs) and moved forward to identify potent chemical scaffolds rather than a single compound. This enrichment strategy based on unsupervised chemoinformatic clustering has been proven helpful in a previous report (Chan et al., 2017). Finally, they discovered a lead compound from the largest chemical scaffold, which suppressed the production of CXCL8, CXCL1, CCL2, and IL-6 from rheumatoid arthritis synovial fibroblasts but not MMP-3. Interestingly, the lead compound acted synergistically with GCs, indicating the possibility of dose-sparing effects of GCs.

Regarding the anti-inflammatory mechanism of Southeast Asian folk medicine, Frutescone O (Fru), Lin et al. proposed several modes of action based on the evidence using LPS-stimulated RAW 264.7, mouse macrophages. The authors discovered that Fru suppressed the production of NO via the downregulation of iNOS, and suppressed the expression of Myd88 resulted in the attenuation of NF-κB pathway and MAPK pathway. Interestingly, Fru interacted with the amino acids of TLR4-MD2, forming one hydrogen bond as well as the other TLR4 antagonist (Gao et al., 2020). Although it was still unsolved which mechanism was responsible, these findings demonstrate the anti-inflammatory effects of Fru. The authors provided evidence for the broader usefulness of Fru which could be shown in several disease models in mice.

## MicroRNA Dependent Regulation of NLRP3 Inflammasome in the MSU- Induced Inflammation

Zhang et al. revealed the negative regulation of NLRP3 inflammasome by miR-223 in RAW 264.7 with miR-223 mimic or inhibitor transfection. During the acute gout attack induced by MSU, inflammasome components, including NLRP3 and ASC, were upregulated as well as the inflammatory cytokines. However, the expression of miR-223 was inversely changed after the MSU stimulation. The transfection of miR-223 mimic or inhibitor attenuated or enhanced the production of inflammatory cytokine production and the components of NLRP inflammasome complex, respectively. These findings provided a novel insight into miR-223 as a negative regulator in acute inflammation.

## A Double-Edged Sword: Immune Response Against Epidemic Coronavirus Infection

Majumdar and Murphy described the ongoing pandemic and the chemokines involved in coronavirus infection, comparing SARS-CoV2 with the previous two coronaviruses, SARS-CoV1 and MERS. COVID-19 is caused by the infection of SARS-CoV2 and can involve complications such as: acute respiratory distress syndrome (ARDS), thrombotic events, multisystem inflammatory syndrome in children (MISC-C), and cytokine storm. Similar to other infectious diseases, promoting the antiviral host defense is beneficial for viral clearance in COVID-19. However, these life-threatening complications might be induced as a result of the dysregulation of the immune system.

Despite the minor differences among the three epidemic coronaviruses, all may share a common pathway to induce ARDS. These viruses primarily infect alveolar epithelial cells and induce inflammatory mediators, including chemokines, to recruit and activate immune cells in the lungs. The activated immune cells further secrete the inflammatory cytokines and chemokines to create a vicious cycle, resulting in the development of ARDS and systemic cytokine storm. Although overall mortality is much lower in COVID-19 (<1%) compared to SARS (10%) or MERS (34%), once the patients reach critical disease severity, the course is similar in all three conditions. Observations from the clinic and animal models of COVID-19 are still being made to identify new targets for therapeutic development.

## The Future Direction of Chemoattractant Targeting Therapy in Neuroimmunological Diseases

Neuroinflammation can occur in infections and autoimmune diseases. Neurological complications have commonly been observed in COVID-19 patients (Varatharaj et al., 2020). Evidence is mounting that in a large proportion of cases, the neurological symptoms do not stem not from direct CNS viral invasion, but from para-infectious mechanisms (Meinhardt et al., 2021; Solomon, 2021). The beneficial effects of dampening the immune response (e.g. with steroids and/or signaling blockade such as anti-IL-6R) indicate that the dysregulated immune system is contributing to pathology. Certainly, a fine balance is needed for maintaining a successful anti-viral response and some severe cases have shown no benefit from immune inhibition (Gupta and Leaf, 2021). As Majumdar and Murphy point out—a complicated signaling network orchestrates immune involvement at different stages and different chemokines attract different cells—including CXCL1/2 for neutrophils, CCL2 and CCL5 for monocytes, and all of the aforementioned and others play a role in T cell activation. The cells can play a protective role in viral clearance, but can tip towards pathology when they cause excess damage and infiltration, especially in an area as sensitive as the central nervous system.

In HSV encephalitis, the IL-1 induction of CXCL1 production by astrocytes and neurons and subsequent neutrophil migration into the brain has highlighted the CXCL1-CXCR2 signaling pathway as a therapeutic target (Michael et al., 2016; Michael et al., 2020). Other chemokine antagonists are already being clinically assessed in the context of stroke, cancer, influenza, and asthma (NIH reporter). Interesting parallels exist amongst neuroinflammatory diseases caused by dysregulation of an immune response outside of the central nervous system—such as in CAR-T-associated encephalopathies and influenza- or tuberculosis-associated encephalitis which also involve cytokine storms and have been shown to respond to anti-IL-6R and other immunosuppressive treatments (Wang and Han, 2018). An interesting case report of a person with neurological complications from SARS infection showed elevated CXCL9 expression in the brain and this warrants further research (Xu et al., 2005).

## The Future Direction of Chemoattractant Targeting Therapy in Rheumatic Diseases

Advances in novel drug application and sophistication of therapeutic strategies in rheumatic diseases, especially rheumatoid arthritis (RA) has improved the quality of disease management and resulted in a decrease in mortality and morbidity. In fact, biological therapies and goal-oriented therapeutic strategies have revolutionized treatment for RA (Aletaha and Smolen, 2018). However, more than half of patients will not reach complete remission using current therapeutic agents (Smolen et al., 2018). Therefore, development of new RA therapies is still needed.

Chemoattractant-mediated leukocyte recruitment is required for igniting inflammation in RA. Thus, chemoattractant targeting therapies will allow us to provide another pathway for attenuating joint inflammation in addition to traditional therapies for RA. While early clinical trials aiming at selective chemokine inhibition failed mostly due to the redundancy of the chemokines, several recent clinical trials targeting the inhibition of CCR1, CXCL10, and CX_3_CL1 demonstrated favorable results, suggesting that there remains an ongoing need to identify the responsible chemoattractants in RA pathogenesis. (Miyabe et al., 2019a).

More recently, C5a receptor inhibitor, avacopan, demonstrated excellent efficacy in the clinical trial in ANCA-associated vasculitis (AAV) (Jayne et al., 2021). Although the mechanism of action in human AAV remained unclear, the inhibition of leukocyte recruitment could be considered to be the central component since C5a acts as a strong chemoattractant. Of note, avacopan combine with immune cell depletion therapy using cyclophosphamide or rituximab was superior to prednisone taper concerning sustained remission, suggesting the proof-of-concept of the next-generation therapeutic strategy for the inflammatory disease (Jayne et al., 2021).

Although no clinical trials have yet been attempted to assess drugs that directly target chemoattractants and/or their receptors in patients with systemic lupus erythematosus, scleroderma and idiopathic inflammatory myositis, numerous chemoattractants and their receptors have been shown to be involved in the recruitment of leucocytes into inflamed organs in those rheumatic diseases and are thus promising targets for therapeutic intervention as well as RA and vasculitis (Miyabe et al., 2019a).

## Summary

Since immune cell recruitment is a fundamental component of inflammation, the chemoattractant system is a promising target in inflammation. The amazing success of avacopan in AAV might indicate the potency of cell-recruitment targeted therapy. Because of the redundancy of chemoattractants, the therapeutic target should be considered carefully. Recent advances in omics analysis in inflammatory disease might enable us to visualize the chemokine system with much fine resolution to identify targets for future drug development.
